# Proximity Begins with a Smile, But Which One? Associating Non-duchenne Smiles with Higher Psychological Distance

**DOI:** 10.3389/fpsyg.2017.01374

**Published:** 2017-08-10

**Authors:** Yevgen Bogodistov, Florian Dost

**Affiliations:** ^1^Frankfurt School of Finance & Management Frankfurt, Germany; ^2^Department of Marketing and Management, European University Viadrina Frankfurt, Germany

**Keywords:** Duchenne smile, politeness, construal level theory, stereotype activation, implicit association test

## Abstract

This study reveals that Duchenne (genuine) and non-Duchenne (non-genuine, polite) smiles are implicitly associated with psychological proximity and distance, respectively. These findings link two extensive research streams from human communication and psychology. Interestingly, extant construal-level theory research suggests the link may work as smiles signaling either a benign situation or politeness, resulting in conflicting predictions for the association between smile type and psychological distance. The current study uses implicit association tests to reveal theoretically and empirically consistent non-Duchenne-smile–distance and Duchenne-smile–proximity associations for all four types of psychological distance: temporal, spatial, social, and hypothetical. Practically, the results suggest several useful applications of non-Duchenne smiles in human communication contexts.

## Introduction

Imagine a politician at a debate on immigration offering abstract stereotypical representations of immigrants and psychologically distant symbolic meanings such as “others,” “them,” or “far away.” Now imagine an educator discussing immigration in class offering concrete individual representations of social minorities and psychologically proximal symbolic meanings such as “us,” “together,” or “familiar.” Both want their communication to match their audience’s psychological distance—that is, how distant, abstract, and stereotypical or how proximal, concrete, and individually detailed information and perceptions are processed (Trope and Liberman, 2010). Furthermore, both communicators might aim to convey a positive appearance with frequent smiles, because doing so can help them obtain a positive perception and attributions of warmth and trustworthiness (Imada and Hakel, 1977; Verser and Wicks, 2006; Nagel et al., 2012). Should they smile in a genuine manner, or should they smile professionally, in a slightly forced way?

Human smiles can be genuine, easily recognized by most observers as a “Duchenne smile.” In contrast, “non-Duchenne smiles” appear in the mouth but not the eyes and are recognized as non-genuine, often polite smiles. (Duchenne de Boulogne, 1862; Ekman et al., 1990, 2002; Ekman and Davidson, 1993; Frank et al., 1993; Manera et al., 2011). Substantial research has investigated the Duchenne smile (for overviews, see Frank and Ekman, 1993; Soussignan, 2002; Gunnery and Hall, 2015); for communication purposes, most studies would recommend a Duchenne smile over a non-Duchenne smile to convey happy emotions (Soussignan, 2002; Schwarz, 2011). Furthermore, studies show that observers rate people showing a Duchenne smile as more persuasive than people showing a non-Duchenne smile (Gunnery and Hall, 2014). The current study furthers this literature by exploring how Duchenne and non-Duchenne smiles directly relate to psychological distance, thus offering a novel perspective on the usefulness of non-Duchenne smiles.

Specifically, we suggest that a Duchenne smile is implicitly associated with psychological proximity, whereas a non-Duchenne smile is associated with psychological distance. Construal-level theory (CLT; Liberman and Trope, 2008; Trope and Liberman, 2010) posits that psychological distance induces abstract (versus concrete) processing of information. Abstract construal levels shift perception focus to general, central, stereotypical aspects and fewer details, whereas concrete construal levels draw attention to individual detail and consideration of peripheral aspects (Liberman and Trope, 1998). Furthermore, abstract construal levels lead to an overgeneralization of perceived social psychological distance as well as other psychological distances such as spatial, temporal, and hypothetical (Bar-Anan et al., 2006; Trope and Liberman, 2010). Researchers have related a plethora of perception effects and behavioral consequences to such changes in construal level and psychological distances (for a recent meta-analysis, see Soderberg et al., 2015). If different smiles could relate to different psychological distances—and thus construal levels—of an audience, knowledge about this effect provides a tool for communicators to support their intended messages subtly. Our two hypothetical smiling communicators, for example, might use non-Duchenne and Duchenne smiles to bolster their respective messages.

Although no study has contrasted different types of smiles, extant CLT research provides two conflicting expectations for the effect of a Duchenne smile on psychological distance. One line of research argues that politeness cues (including a non-Duchenne smile) are linked to psychological distance (Stephan et al., 2010), and the other argues that happiness cues (including a Duchenne smile) are linked to psychological distance (Labroo and Patrick, 2009). We reflect on both arguments and the related prior evidence and then use both politeness theory (Brown and Levinson, 1987) and the feelings-as-information perspective (Schwarz, 2011) to hypothesize a direct and implicit link between Duchenne smiles and psychological proximity and between non-Duchenne smiles and psychological distance.

## Theoretical Background

Psychological distance is itself directly and implicitly interrelated with construal levels (Bar-Anan et al., 2006). A more distant object or event is construed more abstractly and less concretely; for example, one mentally represents a vacation next year in broader, more abstract terms (e.g., destination) than a vacation starting tomorrow (e.g., listing items to remember to pack). The same relationship also works in the other direction, with more abstract mental representations being perceived as more distant. Thus, abstract construal levels overgeneralize to all types of psychological distances, and any specific psychological distance influences all other psychological distances—temporal, spatial, social, and hypothetical (Trope and Liberman, 2010). Psychological distance and related construal level have been shown to play a role in many perception and behavior contexts (for an overview, see Trope and Liberman, 2010) and are increasingly used in human communication (e.g., Nan, 2007; Katz et al., 2016) and emotion (e.g., Gasper and Clore, 2002; Beukeboom and Semin, 2005) studies.

In the context of the impact of smiles on psychological distance, however, we find two conflicting predictions. Labroo and Patrick (2009) posit that a smile acts as a cue of positive mood, signaling a benign situation, such that people feel safe distancing themselves psychologically from it. In contrast, a frowning face signals negative mood, indicating a non-benign situation and forcing people to get psychologically closer and pay attention. These authors empirically demonstrate the effect using smiling and frowning face bullet points as visual stimuli. Transferring this line of argument to Duchenne and non-Duchenne smiles, one would predict that a Duchenne smile signals a happier mood, indicating a more benign situation, such that it should be more strongly associated with psychological distance (or abstract construal level) than a non-Duchenne smile. Nevertheless, a more recent conceptual replication of Labroo and Patrick’s (2009) study demonstrates that various negative emotional stimuli, despite signaling less benign situations, can lead to psychological distance comparable to a positive stimulus (Chowdhry et al., 2015); these authors thus conclude that not all cues less positive than a smiling face bullet point relate to psychological proximity.

However, this line of argument does not address more nuanced situations, such as considering non-Duchenne smiles instead of frowning face bullet points—signals of more or less positive mood indicating more or less benign situations; it may be misleading because “not all smiles are created equal” (Frank and Ekman, 1993, p. 9). People may correctly distinguish clearly happy and unhappy facial cues, which allows them to recognize benign situations, but when asked to recognize a specific emotion from a specific smile, accuracy deteriorates (Ekman, 2006). Participants in smile recognition tasks typically use Duchenne markers to infer a person’s actual enjoyment, independent from his or her emotion (Manera et al., 2011). When discerning the reason behind a non-Duchenne smile, it is necessary to consider the many possible functions of smiling (Niedenthal et al., 2010): People smile not only when they are happy and because a situation is benign, but also when trying to hide embarrassment (Kraut and Johnston, 1979), uncertainty (Labarre, 1947), or sadness (Klineberg, 1940), as well as when they seek power in their communication (Hecht and LaFrance, 1998). Thus, from the feelings-as-information perspective (Schwarz, 2011), a non-Duchenne smile is not simply a display of some less positive emotion. Rather, whereas a Duchenne smile offers a clear signal of positive emotion (Ekman, 2007), a non-Duchenne smile is ambiguous and obscures perception of positive or negative emotion (Prkachin and Silverman, 2002). An unexpected smile may even heighten the ambiguity of the entire communication context (Palomares, 2008). Such uncertain and ambiguous information and stimuli are typically construed more abstractly than certain information, which implies greater psychological distance. When encountering a non-Duchenne smile, people typically focus on the “why” dimension of the context (i.e., higher psychological distance; Liberman and Trope, 2008), because the reasons for a non-Duchenne smile span a wide range of possible explanations, all of which are masked by the smile itself. These arguments support a direct association between the more ambiguous signaling of non-Duchenne smiles and psychological distance (or an abstract construal), and between the more certain signaling of Duchenne smiles and psychological proximity (or a concrete construal).

In addition, a non-Duchenne smile can also signal politeness. People can use a polite smile to mask negative emotions (Ekman et al., 1988; Morse and Afifi, 2015), to meet social demands (Ekman and Friesen, 1982), or to communicate appeasement (Papa and Bonanno, 2008). Politeness has a social meaning and, following politeness theory, directly regulates and reflects social distance (Brown and Levinson, 1987). In other words, politeness cues induce social distance. Recall that according to CLT, this social distance overgeneralizes to other forms of psychological distance (Trope and Liberman, 2010). Stephan et al. (2010) affirm this link between politeness and social, spatial, or temporal distance using several experiments with verbal politeness stimuli and measures. In the present smile context, their findings also predict a direct association between non-Duchenne smiles and psychological distance, and consequently between Duchenne smiles and psychological proximity. Considering all smile- and CLT-related arguments jointly, we expect a direct association between a non-Duchenne smile and psychological distance (or an abstract construal), and between a Duchenne smile and psychological proximity (or a concrete construal). We propose four hypotheses accordingly:

*Hypothesis 1.* Duchenne smiles are associated with low spatial distance, whereas non-Duchenne smiles are associated with high spatial distance.
*Hypothesis 2.* Duchenne smiles are associated with low temporal distance, whereas non-Duchenne smiles are associated with high temporal distance.
*Hypothesis 3.* Duchenne smiles are associated with low social distance, whereas non-Duchenne smiles are associated with high social distance.
*Hypothesis 4.* Duchenne smiles are associated with low hypothetical distance, whereas non-Duchenne smiles are associated with high hypothetical distance.

## Materials and Methods

### Implicit Association Measurement

We tested our hypotheses using implicit association tests (IATs; Greenwald et al., 1998, 2002), a reliable method for uncovering implicit associations between concepts on a deep psychological level. These tests are also commonly used in human communication studies—for example, to reflect stereotype associations (e.g., Arendt, 2013; Kroon et al., 2016).

In an IAT, participants sort the stimuli representing four concepts (Duchenne and non-Duchenne smile pictures; proximal and distal words) into two combined response categories (e.g., Duchenne–proximal, non-Duchenne–distal). We predict that the concepts “Duchenne smile” and “psychological proximity,” as well as the concepts “non-Duchenne smile” and “psychological distance,” are more strongly associated with each other than are the reverse Duchenne smile–distance and non-Duchenne smile–proximity combinations. These two different combinations, thus, constitute the congruent and incongruent conditions for the IAT. The test measures the response time differences required for sorting stimuli in a congruent combined category condition (the hypothesis) and in an incongruent condition. Shorter response times reflect stronger implicit associations; participants are asked to perform the sorting as fast as they can, such that the results reflect non-deliberate and genuinely held associations between concepts, rather than the desired social distance, which explicit measurement tasks are more likely to reflect. Furthermore, by testing combined concepts, the IAT demonstrates robustness to general processing differences in separate concepts. For example, Amit et al. (2009) show that verbal (symbolic distance cues) or visual (smiles) processing mode can directly interact with psychological distance regarding processing time; in contrast, an IAT measures the processing time for combined concepts—distance stimuli and respective smiles—and how easy or difficult it is to mentally associate them. Thus, IATs can provide an effective test for our hypotheses because they are unbiased by desired response behavior and reflect a deep psychological level (Bar-Anan et al., 2006).

### Sample

Our sample consisted of 95 business school undergraduates and 10 business school graduate students (61 women, 44 men) who received course credit for participating in the experiment. Their average age was 22.62 years (*SD* = 4.736). We tested the congruent and incongruent conditions with six smiles and six word pairs for all four types of psychological distance, resulting in 192 trials for each participant.

### Stimuli Materials

**FIGURE 1:**
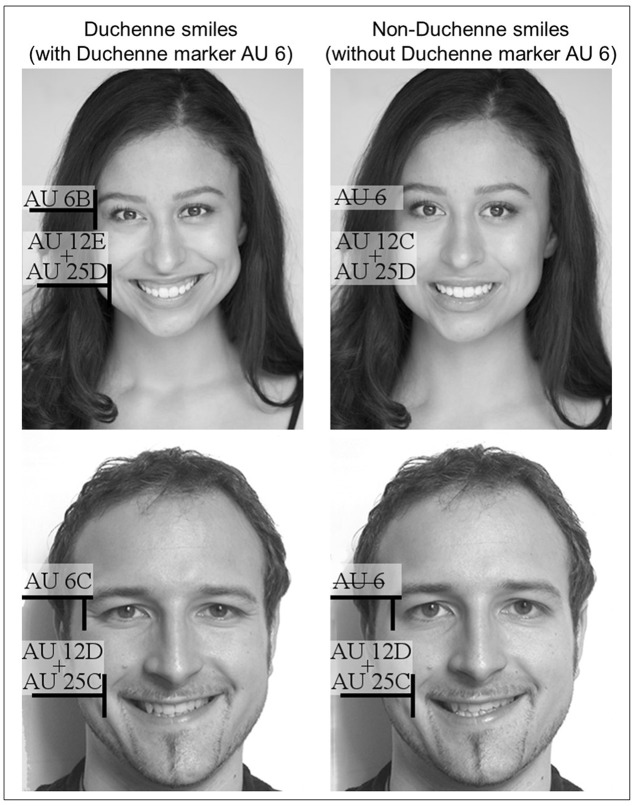
Duchenne smiles and non-Duchenne smiles. Although all smiles show AU 12 and AU 25, only Duchenne smiles show the Duchenne marker AU 6. Woman photographs by Adam Hendershott, who kindly approved publication of the images in this study. Man photographs by Manera et al. (2011).

We selected and tested the stimuli materials using the facial action coding system (FACS; Ekman and Rosenberg, 1997; Ekman et al., 2002). The system proposes a set of action units (AUs) whereby coders define facial expressions. Duchenne smiles contain the so-called Duchenne markers AU 6 (orbicularis oculi, pars lateralis) and AU 12 (zygomatic major), whereas non-Duchenne smiles only show AU 12. Furthermore, AU 6 may differ in intensity from 6A to 6C, and AU 12 from AU 12A to 12E. We specifically selected smile stimuli with predominantly open mouth smiles that showed teeth (AU25) in both Duchenne and non-Duchenne conditions and intensity differences in AU12 of two grades or lower. The presence of AU25 does not affect the intensity of AU12 (Ekman et al., 2002). In total, we collected 12 pictures of three men and three women, with one Duchenne and one non-Duchenne smile each (LaFrance, 2011; Manera et al., 2011), similar to the example in **Figure 1**. Those pictures used in previous academic studies are based on Del Giudice and Colle (2007) smile picture set, and those not taken from extant academic studies were rated by two independent coders using the FACS.

All pictures were converted to grayscale. By varying the JPEG compression, we reduced all images to similarly small (approximately 105 kilobytes) file sizes to guard against systematic differences in loading times. To further eliminate any technical delays, the IAT software routine automatically preloaded all pictures into the cache. Because JPEG compression may reduce the image quality, coders reassessed the pictures to confirm the recognizability of the Duchenne and non-Duchenne smiles, and no recognition concerns arose. For psychological distance, we used six word pairs for spatial proximity and distance and six other word pairs for temporal, social, and hypothetical distance (see the Supplementary Materials for the list of words used). To select these English-language word pairs, we started with obvious terms, such as far–near (spatial distance), past–present (temporal distance), familiar–strange (social distance), and real–unreal (hypothetical distance). Then, we repeatedly looked for antonyms. Finally, we selected the words that most frequently appeared together as synonyms and antonyms. To confirm that the chosen words were not only similar (dissimilar) in meaning but also specific to psychological distances, we conducted a pretest with 25 students in which we asked them to define the type of distance (spatial, temporal, social, or hypothetical, as well as proximal or distal; 4 × 2 = 8 sorting options) associated with the words. Overall, participants agreed with experimenters on which words were proximal and which were distal representations of each psychological distance. The lowest level of agreement was 52% for “lovely” (socially proximal), and the highest was 96% for “inside” (spatially proximal). On average, the level of agreement reached 73.8%. Therefore, we conclude that the words represented the underlying psychological distances as intended.^1^

### Apparatus

**FIGURE 2:**
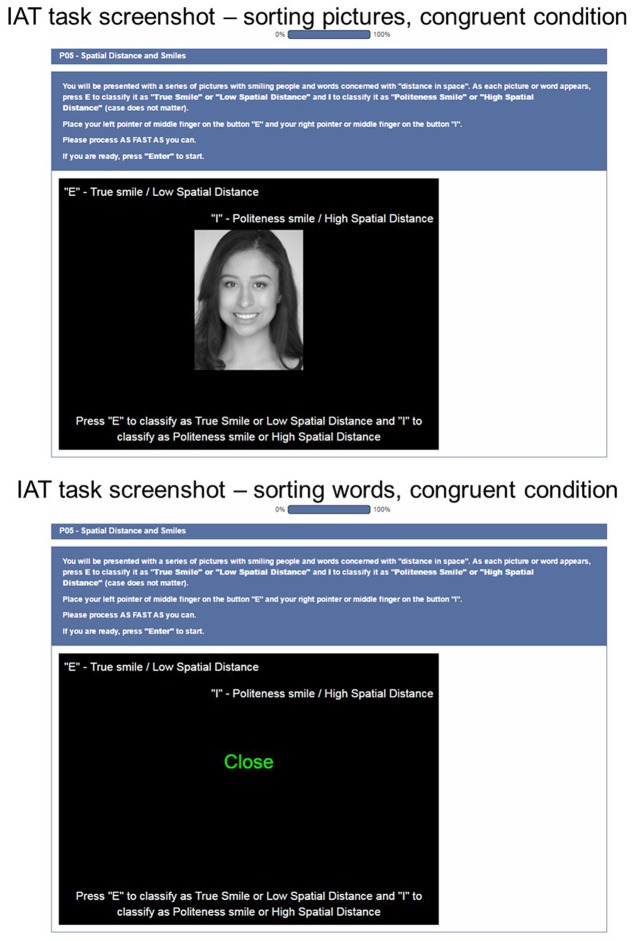
Implicit association test task design. Two screenshots from the first data collection (Block 3, spatial distance). Both show congruent sorting conditions.

Participants viewed all stimuli at 200 pixels × 257 pixels (px) within a black, 700 px × 500 px window on 22′′ screens in 1680 px × 1050 px resolution mode. Stimuli words were presented in green type and category labels appeared in the top left- and right-hand corners in white letters. The viewing distance for all participants was approximately 25′′. **Figure 2** illustrates the setup in a series of screenshots. Participants used their left (right) forefinger to hit the “E” key (“I” key) for left (right) responses on a standard computer keyboard.

### Design

The IAT entails blocks of categorization trials (Greenwald et al., 1998). Block 1 (initial target concept discrimination) included the 6 smile pairs (12 trials total); Block 2 (associated attribute discrimination) included the 6 spatial distance word pairs; Block 3 (initial combined task) was the first data collection block, with 24 smile and word trials in total; Block 4 (reversed target concept discrimination) repeated Block 2 with reversed label position; and Block 5 (reversed combined task) was the second data collection block, repeating block 3 but with reversed spatial distance label positions. Next, we repeated the procedure for the other psychological distances. We dropped the initial target concept discrimination blocks because they remained unchanged throughout the entire study. Thus, we retained 14 IAT blocks in total for the procedure.

To control for learning or order effects, we randomly assigned the students to one of two groups. The first group (*n* = 45) responded to questions in the order of spatial, temporal, social, and hypothetical psychological distances, whereas the other group (*n* = 60) completed the tests in the reverse order.

### Procedure

Groups of 8–16 participants at a time performed the IAT in a computer room, sitting in individual cubicles. An experimenter seated each participant, took student names for course credit, and gave the command to begin. The time between each key press and the next trial was 250 ms. If participants made an error, the word “ERROR” flashed on the screen for 300 ms (added to the trial time), followed by a repetition of the task (Greenwald et al., 1998). Stimuli (words and smiles) were selected randomly (draw without replacement). After participants completed the IAT, they were all debriefed and thanked.

## Results

**FIGURE 3:**
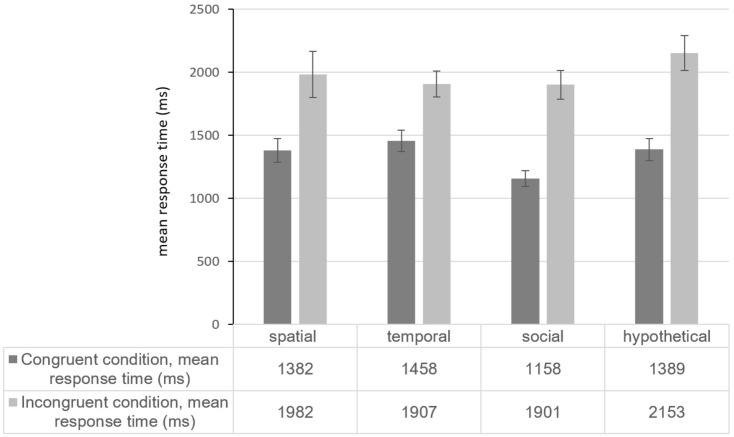
Mean response times by congruent or incongruent condition for four types of psychological distance.

Our hypotheses predicted that Duchenne smiles and proximal words and non-Duchenne smiles and distal words (both the congruent conditions) would produce shorter sorting response times than the incongruent sorting conditions. As shown in **Figure 3**, indeed, participants performed faster when sorting in the congruent conditions than in the incongruent conditions, irrespective of the type of psychological distance.

To test the hypotheses, we then calculated individual effect sizes, called *D* scores—an IAT-specific variant of Cohen’s d. The IAT *D* scores are more robust than mean response time scores to heterogeneous or very fast or slow respondents, which otherwise could distort results with response time outliers (Greenwald et al., 2003). Calculated separately for each participant, *D* is a participant’s mean response time in the incongruent conditions minus the mean response time in the congruent conditions divided by the respective pooled standard deviation of all (congruent and incongruent) response times from that participant. If the hypothesis is correct, then incongruent response times are longer than congruent condition response times, and the *D* score will be positive. Therefore, *D* is typically tested against zero in one-sample *t*-tests. Because this study tests four hypotheses from the same data set, we must apply Bonferroni corrections. Consequently, the corresponding significant Bonferroni-corrected *p*-value is 0.05/4 = 0.0125.

The smallest IAT effect occurred for spatial distance [mean response time difference = 600 ms; *D* = 0.576, *SD* = 0.528; *t*(103) = 11.130, *p* < 0.001, η^2^ = 0.546]. The next smallest effects were temporal distance [mean response time difference = 449 ms; *D* = 0.516, *SD* = 0.423; *t*(101) = 12.320, *p* < 0.001, η^2^ = 0.601] and hypothetical distance [mean response time difference = 768 ms; *D* = 0.733, *SD* = 0.482; *t*(102) = 15.445, *p* < 0.001, η^2^ = 0.701]. The largest effects occurred for social distance [mean response time difference = 743 ms; *D* = 0.801, *SD* = 0.326; *t*(102) = 24.937, *p* < 0.001, η^2^ = 0.859]. All four Bonferroni-corrected tests were significant and confirmed our hypotheses H1–4.

Additional validity and robustness checks posed no serious challenges to our results. No significant learning or order effects arose when we tested the initial group order against the reverse group order (*t* < 1.587). Noting that older respondents might have more social experience to detect a polite smile, we also split the sample at the median age of 22 years; however, we found no difference in *D* scores based on age (*t* < 1.651). Finally, gender can have an influence in emotional perception tasks (Wild et al., 2001), so we compared male and female respondents. We found a weak gender effect for social distance, such that female participants (*D* = 0.856) showed more pronounced IAT effects than male participants [*D* = 0.727; *t*(101) = 2.020, *p* < 0.05, η^2^ = 0.039]. Yet in each subgroup separately, the result directions remained consistent with hypotheses H1–4. Furthermore, the difference is insignificant when applying a Bonferroni correction.

The different sessions and groups of participants did not show any noticeable difference in the results (e.g., caused by naturally occurring changes in context or environment). A multivariate analysis of variance showed no difference in resulting IAT *D* scores between the participant groups [Pillai’s trace = 0.286, *F*(4,44) = 0.630, *p* = 0.969].

In summary, the study confirmed that Duchenne smiles are associated with psychological proximity, whereas polite smiles are associated with psychological distance.

## Discussion

We propose and show that different types of smiles are directly and implicitly associated with different psychological distances. To the best of our knowledge, this is the first study to show an association between smile types—an important feature of human communication in any culture—and a central concept of the increasingly used CLT—psychological distance. Specifically, people associate Duchenne smiles with psychologically proximal concepts, and non-Duchenne smiles regulate social and other psychological distance (Stephan et al., 2010). Previous research shows that this association might itself lead to increased Duchenne smiling: Jakobs et al. (1999) demonstrate, for example, that story-listeners Duchenne smile more if the storyteller is a close friend (i.e., socially proximal) and less with a stranger; they also Duchenne smile more if the storyteller is present (i.e., spatially proximal) and less on the phone. We find that that the association holds in the reverse direction as well: Duchenne smiles lead to psychological proximity.

Our results contrast with Labroo and Patrick’s (2009), who predict that more emotionally positive stimuli (i.e., a Duchenne smile) facilitate more psychological distancing. However, several limitations affecting both the current study and Labroo and Patrick (2009) should be considered to resolve this possible conflict. Labroo and Patrick’s (2009) stimuli (smiling and frowning face bullet points) are already abstract depictions (i.e., symbols) of actual facial expressions. In contrast, the present research uses photographs of actual people smiling in different ways. A generally more abstract depiction might itself prime the participants for an abstract/distant “default” mode of processing (Foerster and Dannenberg, 2010). Then, a frowning face bullet point would simply break with this default abstract processing mode, which could result in the more concrete/proximal processing for the specific cues of negative mood used in Labroo and Patrick’s (2009) studies. In support, Chowdhry et al. (2015) show that the route from positive mood, signaling a benign situation, to abstract construal levels (Labroo and Patrick, 2009) may be oversimplified in that specific negative emotional cues affect psychological distances and construal differently. Investigating the possible role of an abstract versus a more concrete default context on the smile–psychological distance link in the present study could be achieved by replicating the study with more symbolic depictions of Duchenne and non-Duchenne smiles—using cartoons, for example.

Furthermore, future studies could consider using video material instead of still photographs as smile stimulus, though this would preclude using IAT. Videotaped stimuli show the dynamics of the smile—including onset and varying intensities—and more detail, both of which could help establish a concrete construal default context. Videotaped stimuli also generally produce greater effect differences between Duchenne and non-Duchenne smiles than still photographs (Gunnery and Ruben, 2014). Finally, it should be noted that the slight differences in AU intensity between Duchenne and non-Duchenne smiles in the present study do not explain the results. Within Labroo and Patrick’s (2009) framework, a more intense (i.e., Duchenne) smile should associate more with psychological distance, not with psychological proximity as in the present study. A test of response times for the more versus less intense smile pictures (median split) in the initial target discrimination task (IAT block 1) also showed no significant differences, indicating no systematic biases from variation in AU intensities.

The present findings provide researchers with several theoretical and practical implications. First, Duchenne and non-Duchenne smiles offer skilled communicators a subtle means to influence the psychological distance their audiences construe. To date, researchers have treated the two different smiles as showing no significant difference for perceived mood (Kraut and Johnston, 1979; Schwarz, 2002, 2011; Söderlund and Rosengren, 2003): Although people showing a Duchenne smile are rated as more attractive, intelligent, and agreeable than those showing a non-Duchenne smile, both smile types still result in the same perceived trustworthiness (Mehu et al., 2007; Quadflieg et al., 2013). Therefore, Duchenne smiles can help communicators convey a positive image in general, and non-Duchenne smiles can mostly do so while also allowing for psychological distancing and, thus, a more abstract construal. The latter represents a shift in perception with many known and often beneficial consequences. For example, environmental messages such as “Recycle Your Bottles” may be perceived to restrict the freedom of choice; this can be mitigated by inducing a more abstract construal level (Katz et al., 2016). Our study suggests that presenting the message with a non-Duchenne smile would further psychological distance perceptions.

Second, CLT also predicts that judgments at an abstract construal level (or at high psychological distance) reflect the desirable aspects of objects being judged (Liberman and Trope, 1998). For an object with highly desirable aspects, consumers report higher purchase intentions and willingness to pay when primes are more temporally distant (Bornemann and Homburg, 2011; Irmak et al., 2013). Our results suggest an alternative mechanism—using non-Duchenne smiles—that may help a communicator achieve the demanded psychological distance. Moreover, our study is at odds with most extant research recommending the Duchenne smile: Other research shows it can increase tips in service encounters (Bujisic et al., 2014), customer satisfaction (Grandey et al., 2004), and mood (Hennig-Thurau et al., 2006) by signaling positive affect. In contrast, we argue that not just Duchenne smiles lead to positive economic outcomes, because different circumstances dictate different optimal psychological states. Note that recent research increasingly shows that, counter to the longstanding belief that Duchenne smiles can only be generated genuinely, many communicators can display it voluntarily (Krumhuber and Manstead, 2009; Gosselin et al., 2010; Gunnery et al., 2013). These findings bolster our proposal that Duchenne and non-Duchenne smiles can be used as a communication tactic.

Third, those contexts in which psychological distance induced by Duchenne- and non-Duchenne-smile conflict or present a trade-off seem particularly relevant for further research. In terms of effect size, the positive effects of Duchenne smile over non-Duchenne smiles may dominate (a recent meta-analysis estimates an integrated correlation of *r* = 0.39; Gunnery and Ruben, 2014) the psychological distance effects (*r* = 0.23; calculated from Hedges’ *g* = 0.493 in Soderberg et al., 2015). However, the exact behavioral response of a high psychological distance can be unexpected in a social communication context with smiles. For example, experimental participants primed to feel socially excluded (which should be linked to high social distance) not only were more sensitive to the differences in smiles, they were also subsequently more willing to work with a person showing a Duchenne rather than a non-Duchenne smile (Bernstein et al., 2008, 2010). In terms of CLT, one would have assumed a better fit of primed social distance with a (high psychological distance) non-Duchenne smile. It seems possible, but implausible, that the effect is simply the residual from the opposite Duchenne smile and psychological distance effects. Another possibility of interest to further research is investigating whether very high social distance also induces the need to regulate it, a mechanism similar to mood self-regulation. In that case, the self-regulators would likely overcompensate their social exclusion with Duchenne smiling themselves. In summary, the interplay of Duchenne and non-Duchenne smiles confirmed in the present study holds a range of possible behavioral consequences to be explored by future research.

## Ethics Statement

This study was carried out in accordance with the recommendations of the guidelines for research in Brandenburg, Germany, with written informed consent from all subjects. The study neither submits the subjects to emotionally or physically adverse effects, nor to any emotional or physical risk, and as such full ethical review and approval was not required for this study. All subjects were of legal age. All subjects gave written informed consent in accordance with the Declaration of Helsinki. The protocol has been internally reviewed by independent researchers and pre-test subjects and no reasons for requiring an ethics committee approval have been found.

## Author Contributions

All authors listed have made an equal, substantial, direct and intellectual contribution to the work, and approved it for publication.

## Conflict of Interest Statement

The authors declare that the research was conducted in the absence of any commercial or financial relationships that could be construed as a potential conflict of interest.

## Footnotes

1 We tested these English words among German students who understood that they indicated psychological distances. A post-test conversation with these participants indicated that none of the students had any problems understanding the words. Nevertheless, we acknowledge that any future research that uses these words should test them in their own language settings; even though they are common words, participants with different native languages might associate them differently with psychological distances.
