# Supplementary material for: Proximity Begins with a Smile, But Which One? Associating Non-duchenne Smiles with Higher Psychological Distance
Source: Front Psychol. 2017 Aug 10;8:1374. doi: 10.3389/fpsyg.2017.01374 (PMC5554339; doi:10.3389/fpsyg.2017.01374)
Supplement: Supplementary file 1 [file Table_1.DOCX]

Supplementary Material

Proximity begins with a smile, but which one?

True versus polite smiles and psychological distance

Yevgen Bogodistov, Florian Dost^*^

*** Correspondence:**Florian Dost, Assistant Professor of Marketing, European University Viadrina, Große Scharrnstr. 59, 15230 Frankfurt (Oder), Germany
[Dost@europa-uni.de](mailto:Dost@europa-uni.de)

# Supplementary Table

| **Psychological Distance Type** | | | | | | | |
| --- | --- | --- | --- | --- | --- | --- | --- |
| **Spatial** | | **Temporal** | | **Social** | | **Hypothetical** | |
| **Low** | **High** | **Low** | **High** | **Low** | **High** | **Low** | **High** |
| Close | Away | Early | Durable | Authentic | Disliking | Concrete | Abstract |
| Inside | Distant | Immediate | Late | Familiar | Evil | Definitely | Doubtful |
| Internal | External | Present | Long-term | Friendly | Fake | Possible | Imaginary |
| Near | Far | Short-term | Past | Good | Inhabitual | Precise | Impossible |
| Neighboring | Outside | Temporary | Postponed | Lovely | Strange | Real | Uncertain |
| Small | Wide | Today | Tomorrow | Sympathetic | Ugly | Sure | Unreal |

**Supplementary Table 1:** **Stimuli Words for Psychological Distances.**
